# Extracting and Analyzing the S-Parameters of Vertical Interconnection Structures in 3D Glass Packaging

**DOI:** 10.3390/mi14040803

**Published:** 2023-03-31

**Authors:** Jinxu Liu, Jihua Zhang, Libin Gao, Hongwei Chen

**Affiliations:** 1 School of Electronic Science and Engineering, University of Electronic Science and Technology of China, Chengdu 610054, China; 2 State Key Laboratory of Electronic Thin Films and Integrated Devices, University of Electronic Science and Technology of China, Chengdu 610054, China; 3 Chengdu Micro-Technology Co., Ltd., Chengdu 611731, China

**Keywords:** through-glass vias (TGVs), 3D glass packaging, vertical interconnection, transmission matrix (T-matrix)

## Abstract

In order to effectively employ through-glass vias (TGVs) for high-frequency software package design, it is crucial to accurately characterize the S-parameters of vertical interconnection structures in 3D glass packaging. A methodology is proposed for the extraction of precise S-parameters using the transmission matrix (T-matrix) to analyze and evaluate the insertion loss (IL) and reliability of TGV interconnections. The method presented herein enables the handling of a diverse range of vertical interconnections, encompassing micro-bumps, bond-wires, and a variety of pads. Additionally, a test structure for coplanar waveguide (CPW) TGVs is constructed, accompanied by a comprehensive description of the equations and measurement procedure employed. The outcomes of the investigation demonstrate a favorable concurrence between the simulated and measured results, with analyses and measurements conducted up to 40 GHz.

## 1. Introduction

**Figure 1:**
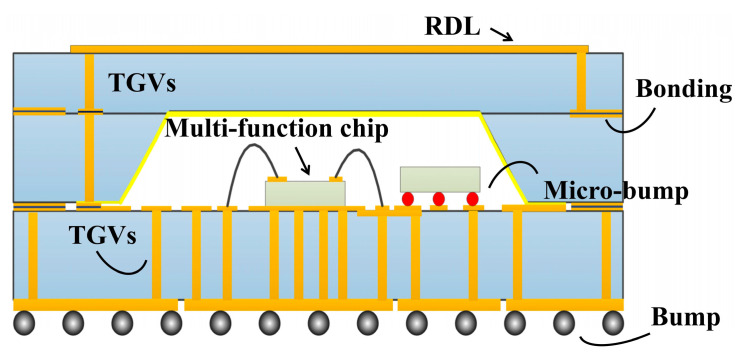
Typical vertical interconnections in glass packaging technologies.

Through-glass vias (TGVs) have garnered increasing attention in packaging technologies owing to their high thermal expansion coefficient [1,2,3,4]. Laser-induced wet etching (LIWE) has emerged as a promising method for low-temperature TGV preparation, offering a potential reduction in surface wave effects [5,6,7,8,9]. As demand for 3D IC applications continues to surge, the quality of vertical interconnections have assumed growing significance, as has their impact on system performance [6]. In order to effectively reduce the insertion loss (IL) introduced by TGVs and enhance the reliability in the high frequency 3D glass packaging design of TGVs structure (as shown in Figure 1), it is necessary to accurately characterize and analyze the high-frequency electrical performance of TGV structures [4]. However, the paucity of published research and the imprecision of S-parameter extraction techniques can compromise the validity of results [10]. Leung et al. [11] used the half-wavelength method to establish a simple short-structure equivalent circuit model and successfully extracted the high-frequency parameters of the through-wafer interconnection reaching 20 GHz. However, this half-wavelength approach is limited by frequency. Moreover, S-parameters extracted from single-port measurements cannot be directly applied to simulations of dual- or multi-port circuits. Ryu et al. [12] employed a dual-port RLCG model to extract S-parameters of through-silicon vias (TSVs) using a de-embedding method with pass-reflection lines (PRL). This involved placing one probe on top of the TSVs and another on a coplanar waveguide (CPW) connected to the bottom of the TSVs. The resulting S-parameters were found to be useful for the integrated simulation of RF circuits [13]. However, if the surface of the TSVs is not flat and there are micro-bumps or bond-wires that do not allow direct contact with the probes, there are difficulties and uncertainties. The most straightforward approach for obtaining vertical interconnections involves contacting the two ends of the interconnection and using plane calibration to extract the S-parameters directly. Unfortunately, in practical applications, it is often observed that the two ends of interconnection are not coplanar, which can pose significant challenges to the testing process.

In this paper, we present an indirect method to extract the S-parameters of two-port TGVs interconnection through the transmission matrix (T-matrix). The method is applicable to any frequency and any structure, including micro-bumps, bond-wires, and various pads. The extracted S-parameters are readily employable in the simulation of 3D integrated circuits.

## 2. Methodology

**Figure 2:**
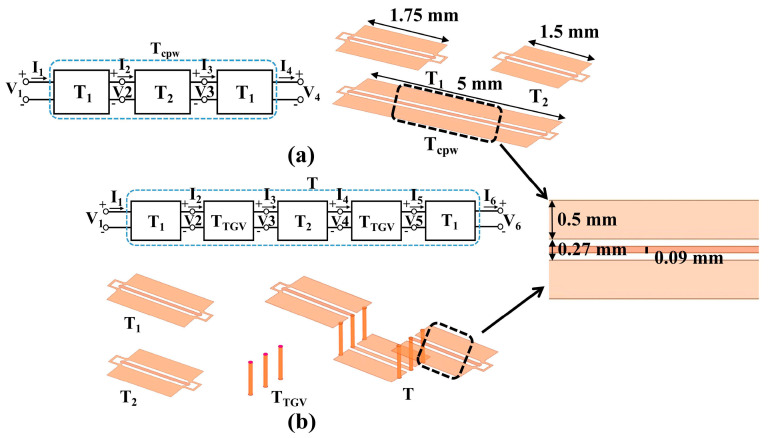
Schematic representation of measure structures needed to perform S-parameter extraction. (**a**) Simulation model of the CPW series without TGVs, (**b**) The CPW series simulation model incorporating TGVs.

In order to facilitate the extraction of the characteristics of interconnection, Figure 2 illustrates the schematic representation of measure structures required for S-parameter extraction. To ensure accurate extraction, two sets of test structures with different CPWs are typically required, *T*_cpw_ and *T*. The *T*_cpw_ structure can be regarded as a cascade of three elements: *T*_1_, *T*_2_, and *T*_1_, used to verify the accuracy of the transmission matrix formula and code. Meanwhile, the *T* structure is a cascade of five elements: *T*_1_, *T*_TGV_, *T*_2_, *T*_TGV_ and *T*_1_, which was used to extract the S-parameters of TGVs in this structure.

The design concept can be described as follows: Figure 2a illustrates the simulation model of the CPW series without TGVs. To thoroughly examine the three constituent parts of the model (*T*_1_, *T*_2_, and *T*_cpw_), we conducted HFSS (EM simulation high-frequency structure simulator) modeling simulations. The material of CPW was determined as gold and the thickness of the glass interposer was set to 550 μm.

Subsequently, we imported the corresponding simulation results (S-parameters) into the MATLAB (Matrix Laboratory, Commonwealth of Massachusetts, USA) program to analyze and validate their accuracy. Essentially, Figure 2a’s simulation model was solely used for verification purposes, whereas the dimensions of all CPW models in this paper are displayed in Figure 2 for ease of comparative analysis. Furthermore, the dimensions of the actual samples were consistent with those of the model.

Figure 2b displays the CPW series simulation model incorporating TGVs. We first obtained the S-parameters through HFSS modeling and by simulating the four parts of the model, namely *T*, *T*_1_, *T*_2_, and *T*_TGV_. We then imported them into the MATLAB program and extracted the S-parameters of TGVs using the Formula (6).

For this cascade configuration, it was more convenient to use the transmission matrix (T-matrix) calculation. Each element was transformed into T-matrix by [14]

(1) $$\left[T_{x}\right]=\left[\begin{array}{cc} A_{x} & B_{x}\\ C_{x} & D_{x} \end{array}\right]=\left[\begin{array}{cc} -\frac{S_{11}S_{22}-S_{12}S_{21}}{S_{21}} & \frac{S_{11}}{S_{21}}\\ -\frac{S_{22}}{S_{21}} & \frac{1}{S_{21}} \end{array}\right]$$

In Figure 2a, the cascade ***T*_cpw_** can be expressed as

(2) $$\left[\begin{array}{cc} A_{T_{cpw}} & B_{T_{cpw}}\\ C_{T_{cpw}} & D_{T_{cpw}} \end{array}\right]=\left[\begin{array}{cc} A_{T_{1}} & B_{T_{1}}\\ C_{T_{1}} & D_{T_{1}} \end{array}\right]\times \left[\begin{array}{cc} A_{T_{2}} & B_{T_{2}}\\ C_{T_{2}} & D_{T_{2}} \end{array}\right]\times \left[\begin{array}{cc} A_{T_{1}} & B_{T_{1}}\\ C_{T_{1}} & D_{T_{1}} \end{array}\right]$$

The S-parameters of ***T*_1_**, ***T*_2_**, and ***T*_cpw_** elements were, respectively, measured and put into Formulas (1) and (2) to verify the feasibility of the mathematical method [15].

In order to extract the S-parameters of TGVs, each element in Figure 2b was converted into a T-matrix using the same method. After conversion, ***T*_TGV_** could be expressed as

(3) $$\left[T_{1}\right]\times \left[T_{TGV}\right]\times \left[T_{2}\right]\times \left[T_{TGV}\right]\times \left[T_{1}\right]=\left[T\right]$$

(4) $$\left[T_{TGV}\right]\times \left[T_{2}\right]\times \left[T_{TGV}\right]={\left[T_{1}\right]}^{-1}\times \left[T\right]\times {\left[T_{1}\right]}^{-1}$$

(5) $$\left[T_{TGV}\right]\times \left[T_{2}\right]=\sqrt{{\left[T_{1}\right]}^{-1}\times \left[T\right]\times {\left[T_{1}\right]}^{-1}\times \left[T_{2}\right]}$$

(6) $$\left[T_{TGV}\right]=\sqrt{{\left[T_{1}\right]}^{-1}\times \left[T\right]\times {\left[T_{1}\right]}^{-1}\times \left[T_{2}\right]}\times {\left[T_{2}\right]}^{-1}$$

Eventually, the S-parameter of TGVs could be extracted. Moreover, a new calibration standard could be developed post-conventional plane calibration. By calibrating the T1 element as part of the main line and transforming *T* into a cascade of three elements, the computational difficulty could be significantly reduced. Considering the complexity and uncertainty of the practical vertical interconnection’s application, we opted for the more intricate case and did not develop a new calibration standard. Extended pads could be calibrated by TRL to move the measurement reference plane to the desired position.

**Figure 3:**
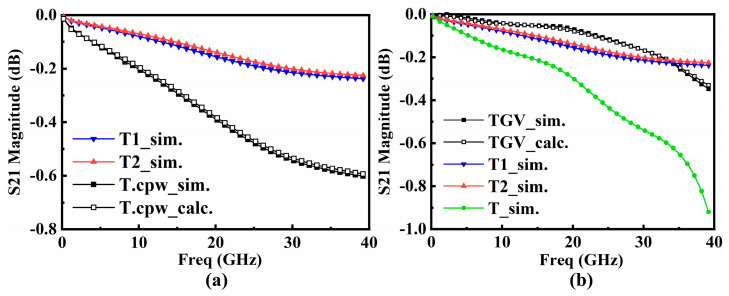
Verification of the validity of S-parameter extraction method from T-matrix. (**a**) Simulation model of the CPW series without TGVs, (**b**) The CPW series simulation model incorporating TGVs.

Subsequently, the actual simulation model was established in HFSS, and the S-parameter results obtained by simulation were imported into the programming calculation software MATLAB for further analysis. By using Formulas (2) and (6), the insertion loss (S21) of both ***T*_cpw_** and ***T*_TGV_** were calculated, as shown in Figure 3. The simulation results were in good agreement with the calculated results, demonstrating the feasibility of the theoretical calculation methods and programming code. The subsequent sections will provide a detailed description of the process and design for extracting the S-parameters of TGVs using these methods. The simulation results demonstrated a high degree of agreement with the calculation results, thereby affirming the feasibility of the theoretical calculation method and programming code. Specifically, Figure 3a shows that the HFSS model simulation result *T.cpw_sim*. and the MATLAB formula code result *T.cpw_calc*., exhibited excellent consistency, indicating the validity of the extraction method and the accuracy of the formulas and codes. Moreover, as illustrated in Figure 3b, the *TGV_sim*., acquired through HFSS simulation, and the *TGV_calc.,* obtained via the MATLAB formula code, also demonstrated a remarkable level of agreement. Consequently, we can use this approach to measure the S-parameters of actual TGVs. However, it is worth noting that some errors exist in the figure, which may be attributed to the boundary condition settings of the simulation. The succeeding sections will provide a comprehensive description of the design and procedure involved in using these methods to extract the S-parameters of TGVs.

## 3. Experimental Procedure and Results Analysis

**Figure 4:**
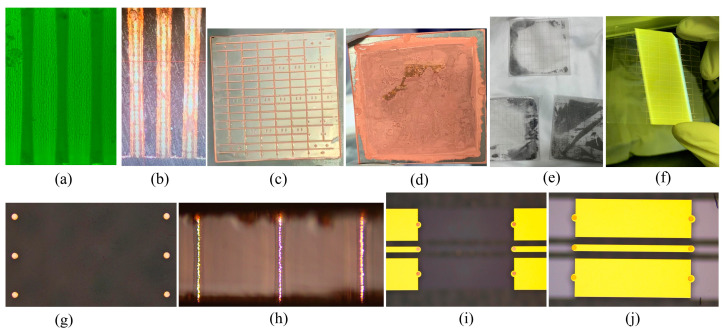
CPW TGVs test structure prototyping process. (**a**) laser-induced corrosion process, (**b**) deep-vias magnetron sputtering to prepare seed layer (copper) process, TGVs electroplating to fill copper process (**c**) front view and (**d**) bottom view, (**e**) grinding to remove surface copper layer process, (**f**) CMP process to obtain smooth TGVs filled with copper interposers (**g**) front-side view micrograph and (**h**) cross-sectional view micrograph, surface gold patterning process (**i**) front view and (**j**) bottom view.

The CPW TGVs test structure prototype process is presented in Figure 4 and verified by proven TGV technology. The experimental steps are as follows: first, 80 μm straight vias were prepared on BF33 glass of 650 μm thickness (dielectric constant and dielectric loss at 30 GHz are 4.8 and 0.007, respectively) via laser-induced wet etching (LIWE) [3]. The titanium and copper thin films are deposited through deep-vias magnetron sputtering, as shown in Figure 4b. Subsequently, the TGVs are fully filled with copper using an electroplating process, as visualized in Figure 4c,d. As excess copper is inevitably electroplated onto the surface of the glass interposer, it is necessary to grind the surface to remove the excess copper, as shown in Figure 4e.

Various methods were utilized during the grinding process, including grinding directly with a machine and manual grinding. However, the application of a conventional magnetron sputtering method to prepare the seed layer may result in inadequate adhesion between the copper layer and the glass interposer surface, leading to complete delamination of the copper during the grinding process. Consequently, the grinder may directly contact the glass, resulting in its shattering. It is noteworthy that the aforementioned phenomena can be avoided if copper-filled TGVs are fabricated through the use of deep-via sputtering and electroplating techniques. This is due to the excellent adhesion of copper deposited through deep-via sputtering, which prevents surface copper from detaching during the grinding process and glass from fracturing.

After grinding, the glass interposer exhibits a highly irregular surface, which is compounded by the presence of a surplus titanium adhesion layer. To remedy this, a chemical mechanical polishing (CMP) process is necessary in order to smooth out the surface of the interposer and simultaneously reduce its thickness to 550 μm. Figure 4f–h underscore the criticality of the CMP stage, as it not only yields a smooth surface on the glass interposer but also in the reduction of its thickness. Furthermore, the copper-filled TGVs are exposed to the atmosphere during the grinding process, resulting in the formation of oxidized copper on their surface. Therefore, the CMP process is vital in eliminating the oxide layer.

Finally, as shown in Figure 4i,j, the test structure of CPW TGVs with 5 μm thick gold was obtained via the use of RDL technique on the glass interposer after the grinding and CMP process. The fabrication process for the RDL layer comprises several key steps, beginning with the sputtering of a 1 μm thick gold seed layer. The desired pattern of CPW-TGVs is obtained via lithography, followed by the production of a 5 μm thick layer of gold through electroplating, resulting in the formation of the final test sample.

**Figure 5:**
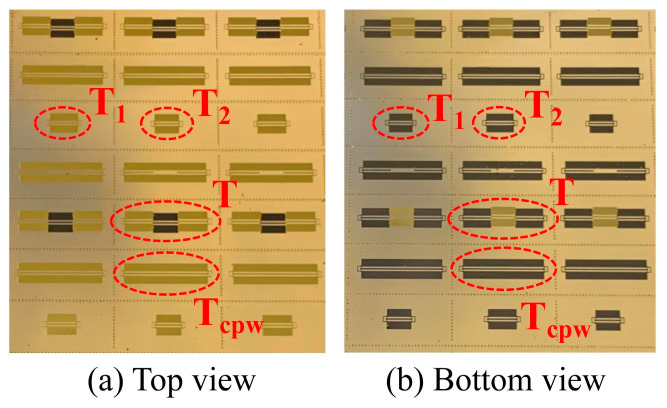
The CPW TGVs test structure prototype. (**a**) Top view and (**b**) Bottom view.

The physical contact between the TGVs and the gold layer was found to be excellent, with no skewing observed. Furthermore, the ground–signal–ground structure exhibited a well-defined 50-ohm impedance. Figure 5 displays the prototype of the CPW TGVs test structure, with the components of each part separated by numerous vias to avoid any electromagnetic interference.

**Figure 6:**
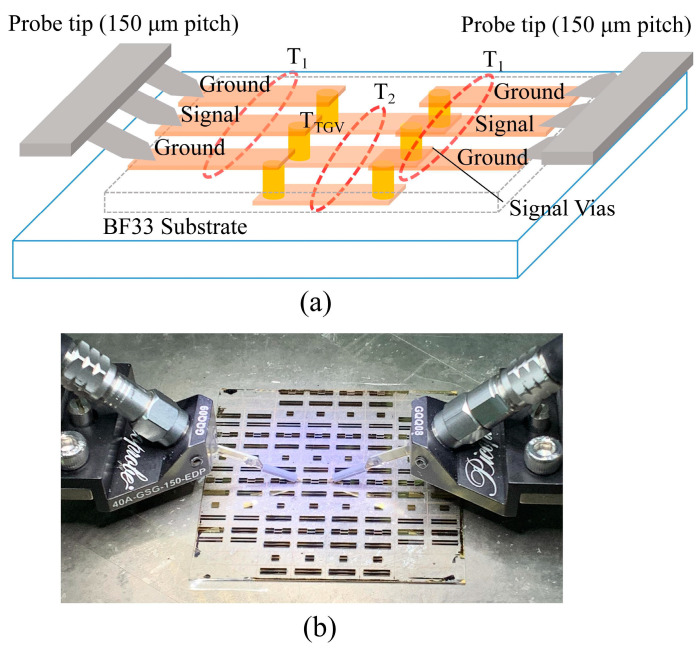
Schematic of the CPW TGVs test structure for S-parameter measurement. (**a**) Model, (**b**) Actual test.

To measure the S-parameters at two ports, two microprobes were positioned at the end of the CPW, as depicted in Figure 6. The S-parameters were measured up to 40 GHz utilizing 150 μm pitch ground–signal–ground (GSG) probes (High-Performance Microwave Probes, Model 40A) connected to a vector network analyzer. The vector network analyzer used for this study was an Agilent PNA N5247A, which is capable of measuring frequencies ranging from 10 MHz to 67 GHz.

**Figure 7:**
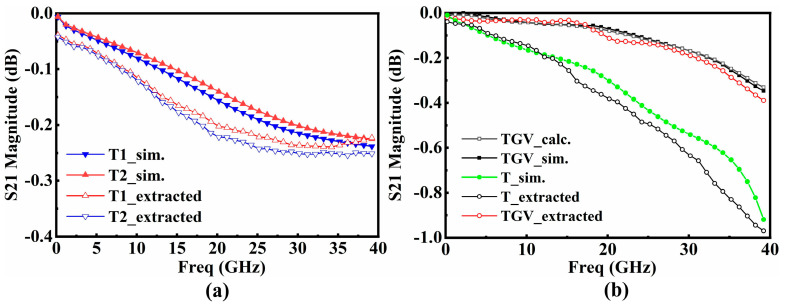
Comparison of the insertion losses measured and extracted of the TGVs with the simulation results. (**a**) Simulation model of the CPW series without TGVs, (**b**) The CPW series simulation model incorporating TGVs.

The S-parameters of ***T*_1_**, ***T*_2_**, and ***T*** were obtained through measurement and the results were imported into MATLAB. The S-parameters for TGVs were extracted through a calculation based on Formulas (1) and (6). The simulation and measurement results are presented in Figure 7. The measured results for ***T*_1_**, ***T*_2_**, and ***T*** were found to be slightly larger, although this trend remained consistent. This discrepancy may be attributed to the HFSS simulation environment and contact errors between the probes and CPW. It is noteworthy that, despite the slightly larger measured results, the TGVs S-parameters extracted through the T-matrix calculation in Section 2 exhibited a high amount of accuracy. This is because the extracted TGVs S-parameter is independent of the actual measurement environment, indicating that this method is highly feasible.

It is also worth noting that the measurement results may be affected by errors that originated from the sample itself. Having conducted an anatomical analysis of the test samples, we have identified the possible causes of these errors and proposed ways to mitigate them.

Firstly, precise control over the diameter of TGVs and the thickness of the dielectric plate is essential. During the actual TGVs fabrication process, errors in the diameter of TGVs can reach up to ±3 μm, while errors in the thickness of the dielectric plate can be up to ±10 μm. These inaccuracies may result from measurement instruments, the inhomogeneity of chemical corrosion solutions, or temperature fluctuations. By effectively managing these process errors, measurement errors can be significantly minimized.

Secondly, controlling the roughness of the inner wall of TGVs is crucial. TGVs are created using laser-induced etching, magnetron sputtering, and electroplating processes to obtain copper-filled TGVs. However, the sidewall of TGVs often has a pit-like rough surface caused by factors such as chemical etchant inhomogeneity, deposits, and air bubbles. During the actual copper filling process of TGVs, the roughness of the copper pillar’s sidewall depends on the sidewall’s roughness. At high frequencies, signal distortion and attenuation can occur due to reflection, refraction, and scattering when the signal flows through the TGVs if the depth of the crater is comparable to the skin depth of copper. By reducing the roughness of the inner wall of TGVs, measurement errors can be effectively reduced.

## 4. Discussion

A T-matrix is presented to extract the S-parameters of vertical interconnection, and demonstrate its efficacy in solving the vertical interconnection problem of glass packaging. The extracted S-parameters exhibit excellent agreement with simulation results, and the proposed method is theoretically capable of addressing vertical interconnection issues in packaging materials across all frequency ranges. In this letter, an example of vertical interconnection in 3D glass packaging was verified and S21 of TGVs up to 40 GHz were successfully extracted.

## Data Availability

Research data are not shared.
